# Comparative transcriptomic analysis of the flower induction and development of the Lei bamboo (*Phyllostachys violascens*)

**DOI:** 10.1186/s12859-019-3261-z

**Published:** 2019-12-24

**Authors:** Yulian Jiao, Qiutao Hu, Yan Zhu, Longfei Zhu, Tengfei Ma, Haiyong Zeng, Qiaolu Zang, Xuan Li, Xinchun Lin

**Affiliations:** 1 State Key Laboratory of Subtropical Silviculture, Zhejiang A & F University, Lin’An, 311300 Zhejiang China; 2 Zhejiang Provincial Collaborative Innovation Center for Bamboo Resources and High-efficiency Utilization, Lin’an, 311300 Zhejiang China; 3 Key Laboratory of Synthetic Biology, Institute of Plant Physiology and Ecology, Shanghai Institutes for Biological Sciences, Chinese Academy of Sciences, Shanghai, 200032 China

**Keywords:** *Phyllostachys violascens*, Flowering, RNA-seq, Plant hormone signal transduction, Stress responsive, MADS box

## Abstract

**Background:**

Bamboo is a very important forest resource. However, the prolonged vegetative stages and uncertainty of flowering brings difficulties in bamboo flowers sampling. Until now, the flowering mechanism of bamboo is still unclear.

**Results:**

In this study, three successive stages of flowering buds and the corresponding vegetative buds (non-flowering stage) from Lei bamboo (*Phyllostachys violascens*) were collected for transcriptome analysis using Illumina RNA-Seq method. We generated about 442 million clean reads from the above samples, and 132,678 unigenes were acquired with N50 of 1080 bp. A total of 7266 differentially expressed genes (DEGs) were determined. According to expression profile and gene function analysis, some environmental stress responsive and plant hormone-related DEGs were highly expressed in the inflorescence meristem formation stage (TF_1) while some floral organ development related genes were up-regulated significantly in floral organs determination stage (TF_2) and floral organs maturation (TF_3) stage, implying the essential roles of these DEGs in flower induction and maturation of Lei bamboo. Additionally, a total of 25 MADS-box unigenes were identified. Based on the expression profile, B, C/D and E clade genes were more related to floral organs development compared with A clade genes in Lei bamboo.

**Conclusions:**

This transcriptome data presents fundamental information about the genes and pathways involved in flower induction and development of Lei bamboo. Moreover, a critical sampling method is provided which could be benefit for bamboo flowering mechanism study.

## Background

In plant kingdom, flowering is necessary for the transition from vegetative stage to reproductive stage as one of the most important process. Investigation into *Arabidopsis thaliana* reveals that the phenomenon of the flowering is controlled by diverse environmental and endogenous factors, such as temperature, light signals, day length and plant hormones [1–4]. For example, plant endogenous hormones Gibberellin (GA) could promote flower development and low temperature will cause flowering delay in *Arabidopsis* [5, 6]. Additionally, the ABCDE model genes are widely used for understanding floral development [7], and the MADS-box genes are well known as ABC model factors to control the floral organ identity during flower development [8]. According to ABC model, B, C and E genes control petal and stamen identity; C and E genes control carpel formation; D and E genes are involved in ovule identity [9–11].

Bamboo is one of the most important non-timber forest resources in the world. Its fast growth and strong nitrogen fixation capacity have received much attention in economy and ecology. However, the prolong vegetative period and the death of bamboo after flowering, lead to great economic and ecological losses [12]. The uncertainty of bamboo flowering time increased the difficulty of sampling, further inhibiting the study of flower induction to bamboo. Fortunately, the completion of moso bamboo (*Phyllostachys edulis*) draft genome sequencing is beneficial to screen the flowering related genes. For example, the transcription factors play an important role in gene expression, thereby influencing the floral transition, the *PheMADS14* and *PheMADS4* obtained from moso bamboo may play vital regulatory roles in flower development [13–16]. Lei bamboo (*Phyllostachys violascens*), widely distributed over south of China, has high economic value because of its bamboo shoots edibility. The income for intensively managed Lei bamboo is about 20 times higher than that of rice [17]. During flowering, however, shoot production decreases sharply. Different from moso bamboo flowering gregariously with 60–120 years period [18], Lei bamboo flowers sporadically [19]. It suggests a similar but not identical flowering mechanism between moso bamboo and Lei bamboo. Recently, some flowering genes in Lei bamboo have also been identified, such as *PvFRI-l*, *PvSOC1* and *PvMADS56* [20–22]. However, the flowering mechanism in Lei bamboo is still poorly understood.

Next-generation sequencing [23] technologies such as the Illumina Solexa, Roche 454, and ABI SOLID platforms have revolutionized biological research by providing genomic and transcriptome data rapidly and inexpensively [24]. In this study, we used RNA-sequencing (RNA-seq) to investigate the flowering mechanism of Lei bamboo by transcriptome analysis [25]. The goal of this study was to obtain a complete set of assembled unigenes and transcripts for Lei bamboo and to identify flowering-related genes. Our data will be useful for Lei bamboo flowering study and provides a critical sampling method to study flowering mechanism in bamboos.

## Methods

### Plant materials

**Fig. 1:**
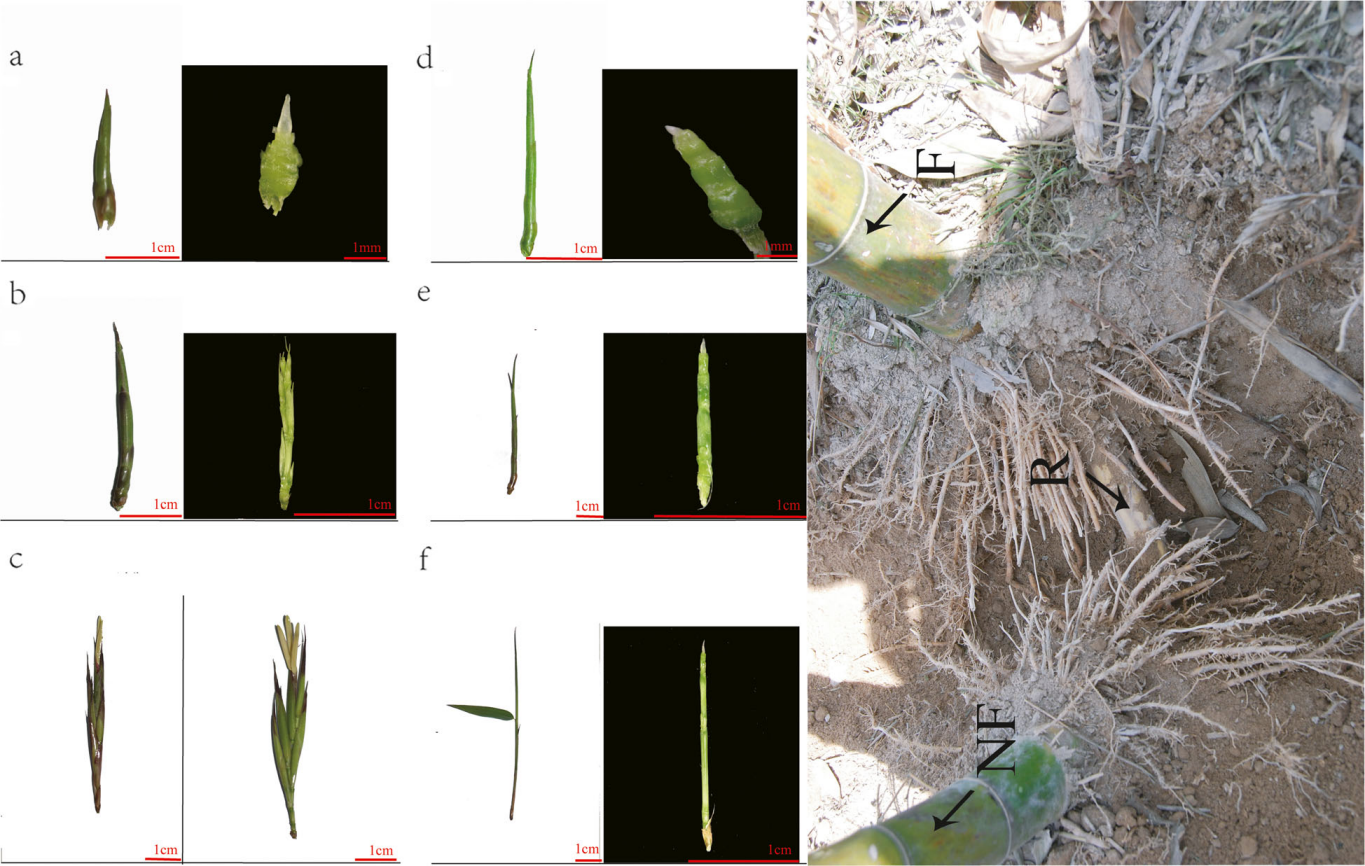
The reproductive and vegetative bud samples of Lei bamboo. **a**-**c** The flowering buds sampled on March 8th, March 29th and April 12th are named as TF_1, TF_2 and TF_3 respectively. **d**-**f** Vegetative buds sampled on March 8th, March 29th and April 12th are named TV_1, TV_2 and TV_3 respectively. **g** The same rhizome of flowering and non-flowering plants

After observing flowering characteristics of Lei bamboo for more than 10 years, we began to learn and pre-judge before flowering occurs with a certain probability [26]. Samples of Lei bamboo were collected from bamboo forests cultivated in Lin’an, Zhejiang province, China. Flowering buds and vegetative buds were collected at about 2 pm from expected flowering and non-flowering plants from the same rhizome, at different stages: March 8th and 29th and April 12th, 2013. The expected bud samples confirmed after bamboo flowering were used for transcriptome sequencing. Flowering buds from a flowering bamboo plant were sampled at three stages, formation of inflorescence meristem (TF_1), formation and identity determination of floral organs (TF_2), and maturation of floral organs (TF_3) stages. Vegetative buds (TV) from a non-flowering bamboo plant growing on the same rhizome of the flowering bamboo plant were both sampled at the same time (Fig. 1g). The first two stages (TF_1 and TV_1) were sampled on March 8th, 2013, when the inflorescence meristem of bamboo began to form, but the meristem morphology of flower buds was similar to that of vegetative buds (Fig. 1a, Fig. 1d). The second two stages (TF_2 and TV_2) were sampled on March 29th, 2013, when the inflorescence appeared, and the floral organs started to take shape (Fig. 1b, Fig. 1e). The third two stages (TF_3 and TV_3) were sampled on April 12th, 2013, when the floral organs continued to develop and gradually become mature (Fig. 1c, Fig. 1f). The six samples were used for cDNA library construction and RNA-seq separately. All samples were transferred to liquid nitrogen immediately and stored in − 80 °C.

### RNA-seq sequencing and transcriptome assembly

We extracted total RNA from samples by using TRIzol reagent (Invitrogen) and treated with RNase-free DNase I (New England Biolabs) to remove any contaminating genomic DNA. Then cDNA libraries were constructed by using NEBNext® Ultra™ Directional RNA Library Prep Kit for Illumina® (NEB, USA) instruction. Raw data (raw reads) in the fastq format were first processed by using in-house perl scripts. To ensure high data quality, we acquired the clean data by removing reads containing an adapter or poly-N and low-quality reads, then the clean-data quality including Q20, Q30, GC-content and error rate were calculated (Table 1). Finally, the clean data was assembled by using Trinity software with min_kmer_cor set to 2 by default; all other parameters were set to the default [27].

**Table 1:** The analysis of data output quality. Sample: Sample name_1, left reads; Sample name_2, right reads. The total number of clean reads is left + right. Q20: the percentage of bases with a phred value > 20. Q30: the percentage of bases with a phred value > 30. GC content: the GC ratio of the total base number

Sample	Raw Reads	Clean reads	Clean bases (G)	Error (%)	Q20 (%)	Q30 (%)	GC (%)
TV_1_1	35,922,083	33,853,965	3.39	0.04	96.64	90.17	52.56
TV_1_2	35,922,083	33,853,965	3.39	0.05	95.45	88.16	52.60
TV_2_1	35,822,179	33,894,300	3.39	0.04	96.64	90.14	52.98
TV_2_2	35,822,179	33,894,300	3.39	0.05	95.46	88.16	53.03
TV_3_1	44,653,016	42,244,063	4.22	0.04	96.63	90.10	53.02
TV_3_2	44,653,016	42,244,063	4.22	0.05	95.41	88.03	53.06
TF_1_1	36,420,947	34,377,877	3.44	0.04	96.51	89.87	52.74
TF_1_2	36,420,947	34,377,877	3.44	0.05	95.26	87.75	52.80
TF_2_1	40,450,621	38,236,522	3.82	0.04	96.58	90.01	52.88
TF_2_2	40,450,621	38,236,522	3.82	0.05	95.44	88.09	52.94
TF_3_1	40,982,518	38,751,791	3.88	0.04	96.70	90.29	52.28
TF_3_2	40,982,518	38,751,791	3.88	0.05	95.57	88.39	52.32

### Analysis of differential expressed genes

Expression level of genes is calculated by using RPKM method [28, 29]. For each sequenced library, the read counts were adjusted by using the edgeR program package with one scaling normalized factor. The DEGseq R package was used for differential expression analyses. Qvalue < 0.05 & |log2 (fold change)| > 1 were set as the thresholds for significantly differential expression. Gene function was annotated by using the following databases: Nr (NCBI non-redundant protein sequences), Nt (NCBI non-redundant nucleotide sequences), Pfam (Protein family), COG (Clusters of Orthologous Groups of proteins), Swiss-Port (A manually annotated and reviewed protein sequence database), KO (KEGG Ortholog database) and GO (Gene Ontology) [30–32]. We used NCBI blast 2.2.28^+^to search genes in the Nr, Nt and Swiss-Port databases with E-value 1e-5, and the COG database with E-value 1e-3 [33]. We predicted the protein domain from Pfam using HMMER 3.0 software and hmmscan with E-value 1e-5 [34]. Blast2GO v2.5 was used for GO function annotation based on the annotation of the NR and Pfam databases [35]. GO enrichment analysis of differentially expressed genes (DEGs) involved in the GOseq R packages based on Wallenius non-central hyper-geometric distribution, when the corrected *P*-value < 0.05, the function is regarded as an enrichment item [36]. We used KOBAS software to test the statistical enrichment of DEGs in KEGG pathways with E-value < 10^− 5^ and rank ≤5 [37].

### Identification and phylogenetic analysis of the MADS-box gene family

The *Arabidopsis* MADS protein sequences were acquired from TAIR (http://www.arabidopsis.org/) database. We used *Arabidopsis* MADS domain sequences as query to blast against the Lei bamboo transcriptome database. Multiple sequence alignment was performed using the full-length protein sequences in the program Clustal W. The un-rooted neighbor-joining (NJ) [23] and maximum likelihood (ML) [38] trees were constructed by using the MEGA6 package [39] with bootstrap support of 1000 replicates.

### qRT-PCR analysis

Several DEGs involved in flower development of Lei bamboo were chosen for validation with quantitative real-time PCR (qRT-PCR). qRT-PCR reaction was analyzed with the CFX96TM real-time PCR detection system (Bio-Rad, Hercules, USA) with the SYBR Green Master Mix (Takara, Dalian, China), and amplified with 1 ul of cDNA template, 10 ul of 2 x SYBR Green Master Mix, and 1ul of each primer (10 umol/ul), to a final volume of 20 ul by adding water. The amplification program consisted of 1 cycle of 95 °C for 30s, followed by 39 cycles of 95 °C for 5 s, TM for 20s, 72 °C for 20s. The *Actin* was used as reference gene. All the qPCR assays were performed with three biological and four technical replicates, and the quantitative analysis used the 2^-ΔΔCT^ method.

## Results

### Identification of total unigenes during Lei bamboo flower development

A total of 468,502,728 raw reads were generated by Illumina sequencing of TF and TV, resulting in 442,717,036 clean reads after moving low quality sequences (Table 1). Assembly of the clean reads produced 894,499,502 unigenes with sequence length range of 201–16,212 bp. The N50 length of unigenes was 1080 bp and the N90 length was 269 bp (Table 2).

**Table 2:** The length distribution analysis

Transcript length interval	200-500 bp	500-1kbp	1 k-2kbp	>2kbp	Total	N50 (bp)	N90 (bp)
Number of transcripts	125,980	64,002	68,737	58,554	317,273	1968	473
Number of unigenes	85,724	24,704	13,221	9029	132,678	1080	269

**Fig. 2:**
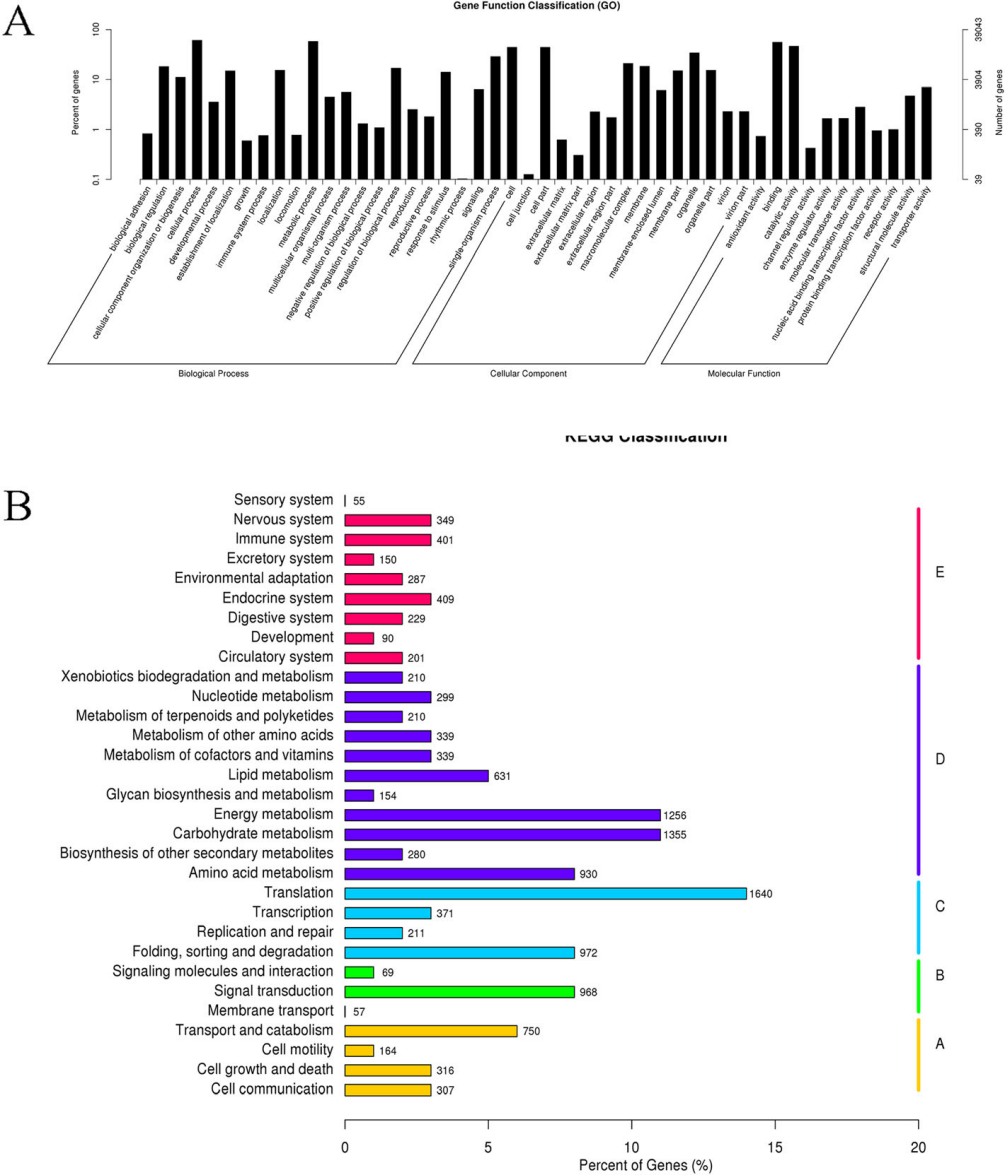
The functional analysis of total unigenes in Lei bamboo. **a** GO functional classification (**b**) KEGG classification

In this study, we analyzed the function of the total unigenes by annotating the unigenes with seven databases (Additional file 1). A total of 2931(2.2%) unigenes were annotated in all databases and 58,628 (44.18%) unigenes were annotated in at least one database. According to the GO analysis (Fig. 2a), the genes associated with “cellular process”, “metabolic process”, “single-organism process”, “biological regulation” were enriched. To identify biochemical pathways, we mapped the annotated sequences in the KEGG database (Fig. 2b). Most unigenes were involved in “translation”, “energy metabolism” and “carbohydrate metabolism”, with the least in “membrane transport”, “signaling molecules” and “degradation”. These pathway assignments provide valuable information for investigating specific biochemical and development processes.

### The phytohormone related and stress responsive DEGs are involved in Lei bamboo flower induction

**Fig. 3:**
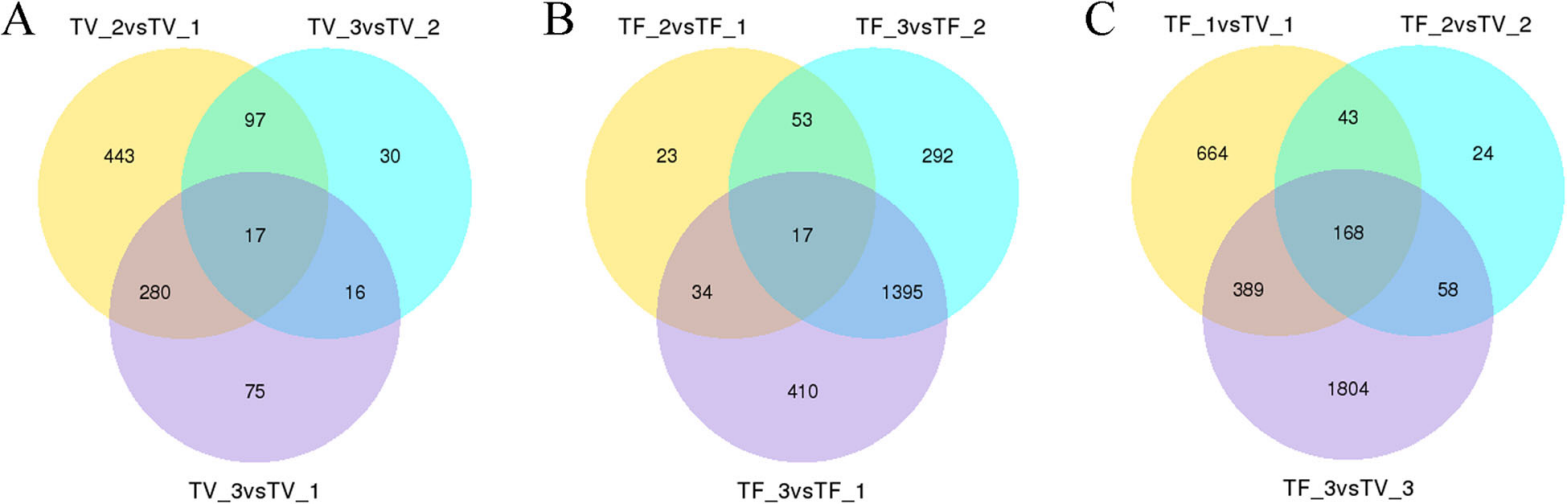
The venn diagram of differentially expressed genes (DEGs) in Lei bamboo. **a** the DEGs in TV stages (**b**) the DEGs in TF stages (**c**) the DEGs between TF and TV stages

A total of 7266 unigenes were identified as DEGs from 16 comparisons (TF_1 vs TF_2, TF_1 vs TF_3, TF_2 vs TF_3, TF_23 vs TF_1, TF13 vs TF_2, TF12 vs TF_3, TV_2 vs TV_1, TV_3 vs TV_1, TV_3 vs TV_2, TV23 vs TV_1, TV_13 vs TV_2, TV_12 vs TV_3, TF_1 vs TV_1, TF_2 vs TV_2, TF_3 vs TV_3, TF vs TV). According to the expression pattern (Additional file 2), more than half of the DEGs were highly expressed at TF_3. In the rest DEGs, most were highly expressed in TV stages, but only a few genes have higher expressions in former stages of flower development (TF_1 and TF_2). In order to better to analyze the flowering mechanism of Lei bamboo, we categorized the samples according to their development stages and analyzed the DEGs involved in TV and TF stages (Fig. 3a and b). For TV stages, 388, 837 and 160 DEGs were identified in the comparisons of TV_3 vs TV_1, TV_2 vs TV_1 and TV_3 vs TV_2, while for TF stages, 127, 1856 and 1757 DEGs were found in the comparisons of TF_2 vs TF_1,TF_3 vs TF_1 and TF_2 vs TF_3. After removing the duplicated ones separately, a total of 958 DEGs in vegetative buds and 2224 DEGs in flowering buds were finally identified.

**Fig. 4:**
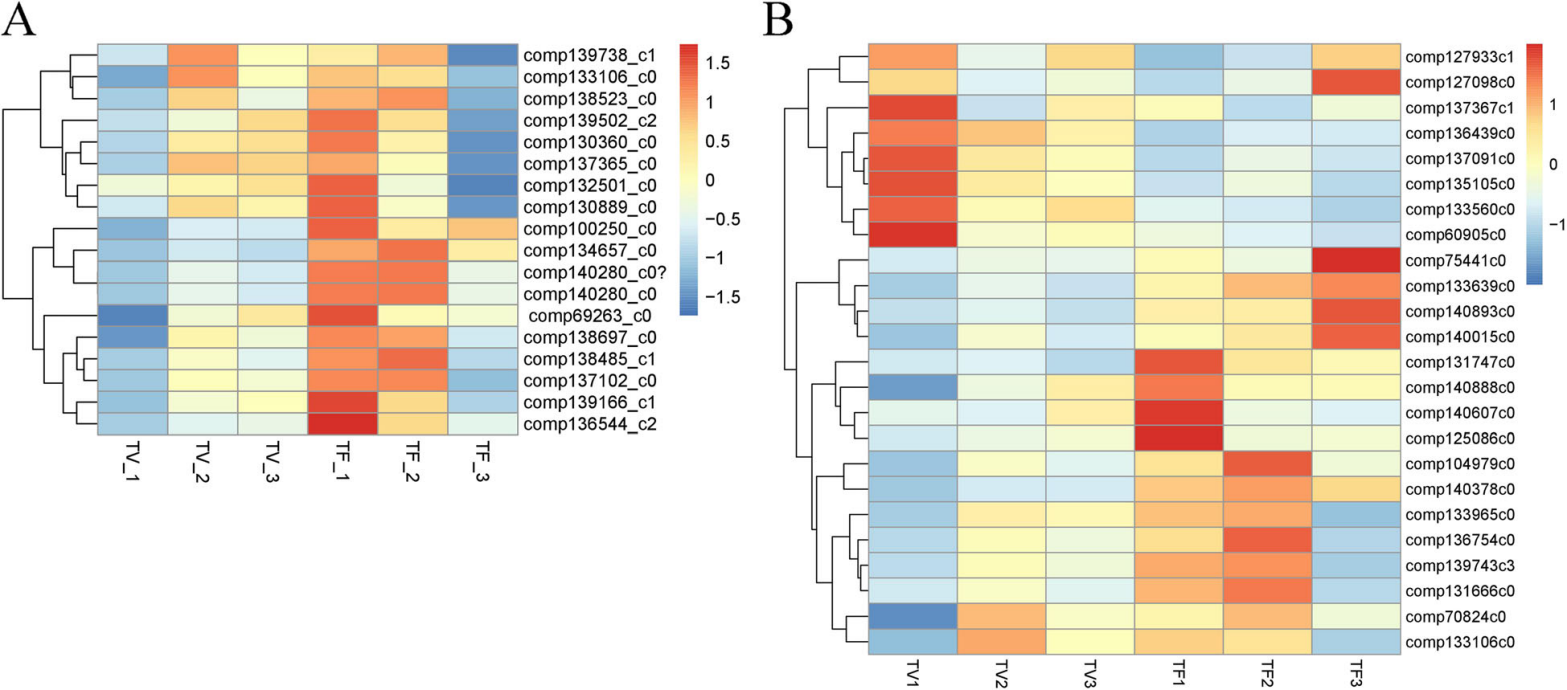
The heatmap of the selected DEGs in Lei bamboo flowering (**a**). the genes involved in plant hormone signaling pathway (**b**) the genes involved in photoperiod and stress resistance

As described before, the samples of TF_1 and TV_1 were collected at the same time when the inflorescence meristem began to form, but there was no flower meristem morphology in TF_1 (Fig. 1). It indicates the TF_1 might be flower induction stage in Lei bamboo. Although the two periods of TF_1 and TV_1 differed in flower induction, they experienced the same biological processes, such as vegetative buds and stems development. Based on KEGG analysis (Additional file 3), the DEGs in TF_1 (compared with TF_2 and TF_3) were enriched in a number of same biological pathways as well as in TV_1 (compared with TV_2 and TV_3) such as the “photosynthesis”, “cell cycle”, “metabolic” and “DNA replication” pathways. To better to obtain the flower induction related genes, we removed the coincident genes in TF_1 compared to TV_1. Finally, a total of 395 genes (28 from the comparison of TF_1 vs TF_2, and 367 from the comparison of TF_1 vs TF_3) were differential expressed significantly specific in TF_1. According to KEGG analysis, the “plant hormone signal transduction” pathway was abundant in TF_1 compared with corresponding TV_1 (Additional file 3) and a serious of highly-expressed related genes (mainly includes *ARF*, *CRE1*, *BRI1*, *BSK*, *NPR1*, *AUX1*, *TGA*, *TIR1*, *B-ARR* and *CTR1*) were identified. The expression profile suggests these plant hormones related DEGs should be involved in the regulating of Lei bamboo flower induction (Fig. 4). Additionally, we found the “detection of abiotic stimulus”, “detection of external stimulus”, “detection of light stimulus” related genes are up-regulated but “long-day photoperiodism, flowering” related genes are down-regulated in TF_1 (compared with TV_1), respectively (Fig. 4b). It reflects that the flower induction of Lei bamboo possibly affected by environmental stress factors. Therefore, the genes involved in stress responsive and phytohormone should be essential for the process of Lei bamboo flower induction.

### Floral organ development related DEGs are involved in Lei bamboo flower maturation

With the development of Lei bamboo flowers (TF_2), the “lipid metabolic and biosynthetic process”, “oxidoreductase activity” related genes were up-regulated (compared with TF_1). They were involved in the “flavonoid biosynthesis”, “circadian rhythm”, “biosynthesis of secondary metabolites” and “metabolic” pathways (Additional file 3 and Additional file 4), indicating their essential roles in formation of floral organ primordia. Moreover, a total of 1149 genes were up-regulated in TF_3 compared with TF_1 and TF_2. In this stage, the floral organ began to mature. GO analysis showed that the “single-organism metabolic process”, “cytoplasmic part”, “catalytic activity” related genes were abundant. They were enriched in a large number of metabolic processes of “Ribosome”, “beta-Alanine metabolism”, “Ascorbate and aldarate metabolism” (Additional file 3 and Additional file 4). It reflects the maturation of flower organs, especially the reproductive organs of carpels and stamens, requires a great deal of material and energy exchange in the TF_3 stage of Lei bamboo.

**Fig. 5:**
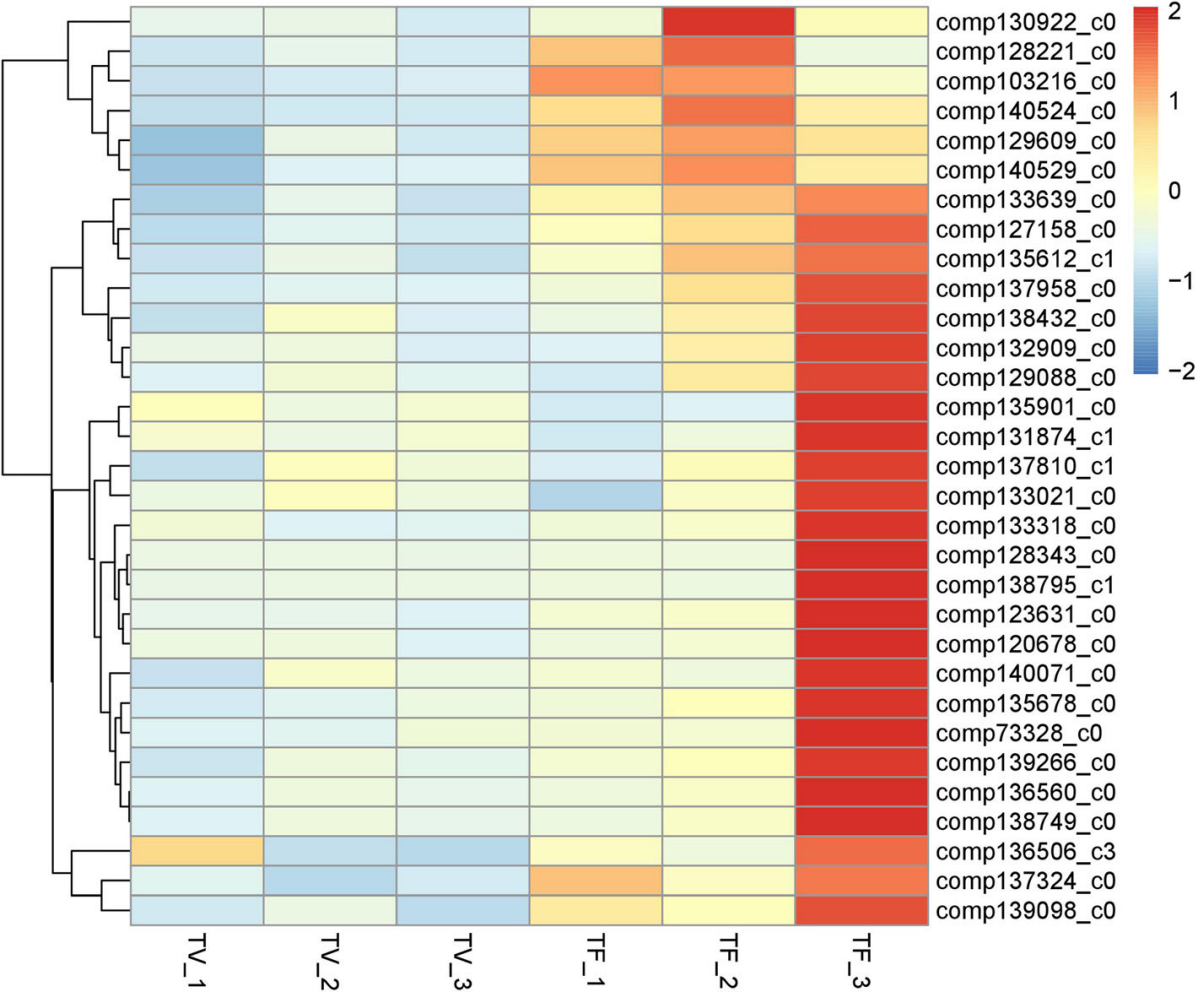
The heatmap of the selected DEGs involved in floral organ development of Lei bamboo

To better determining the critical genes during Lei bamboo flower organ development, a comparative analysis was made between two later reproductive stages (TF_2 and TF_3) with the corresponding vegetative stages (TV_2 and TV_3), and a total of 293 and 2419 DEGs were get. Among the DEGs, 1377 genes were found to be up-regulated during floral organs identity determination (TF_2) and floral organs maturation (TF_3) stages, that were enriched in the functions of “oxidation-reduction process”, “single-organism metabolic process”, “cytoplasmic part” and “catalytic activity”. They were abundant in “beta-Alanine metabolism”, “Ribosome”, “Ascorbate and aldarate metabolism”, “Amino sugar and nucleotide sugar metabolism” pathways. Additionally, some genes involved in “female gamete generation”, “flower development”, “pigment biosynthetic process”, “anther morphogenesis” were found to differential expressed significantly in the latest stage (TF_3), such as *comp135901_c0*, *comp128343_c0*, *comp136560_c0*, *comp136560_c0*, *comp136560_c0* (Fig. 5), reflecting their critical roles in flower organ formation and maturation.

### The B, C/D and E MADS genes are involved in flower organ development of Lei bamboo

**Fig. 6:**
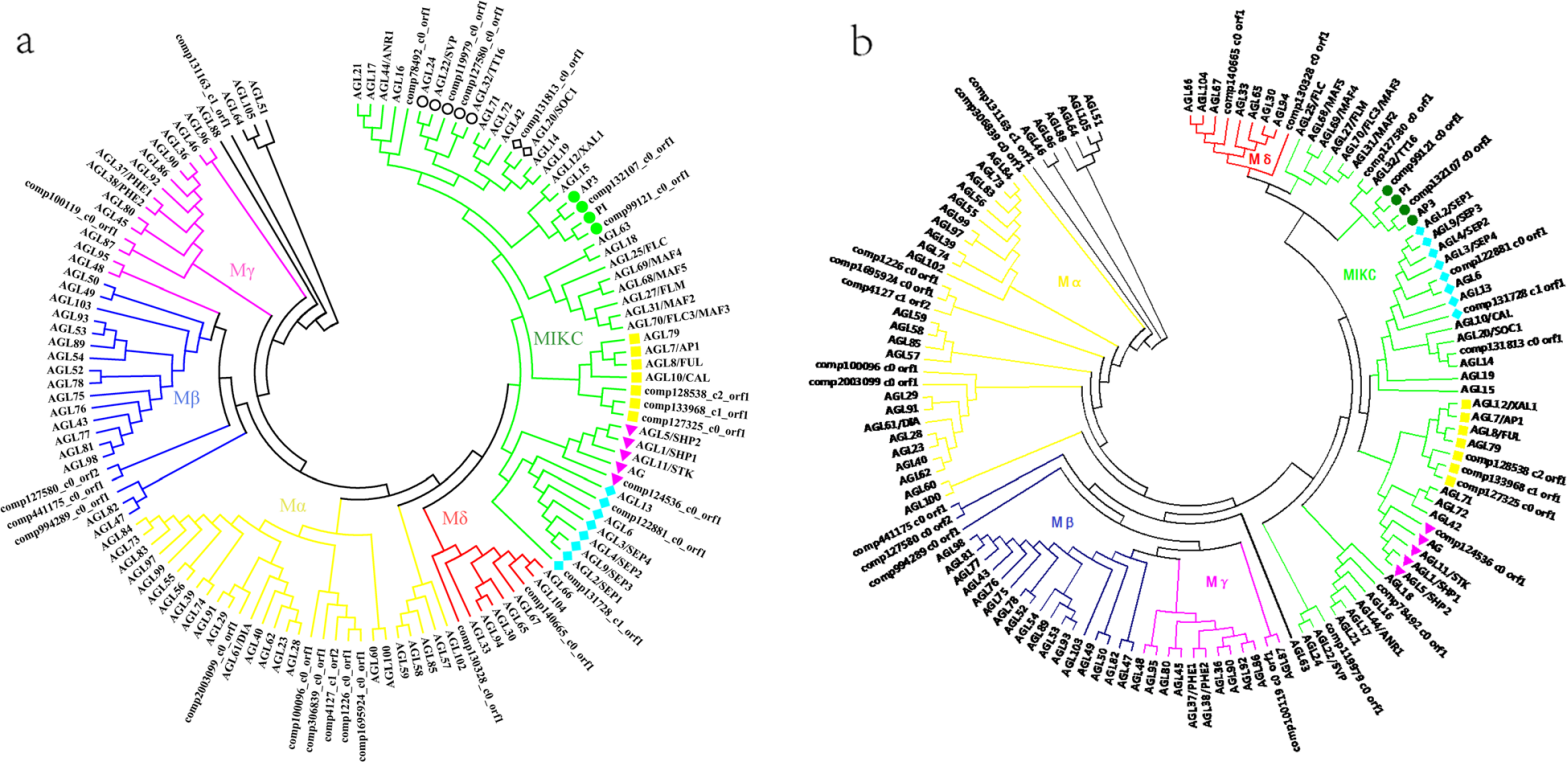
Phylogenetic analysis of MADS-box proteins in *Arabidopsis* and Lei bamboo. **a** A total of 25 open reading frame sequences in Lei bamboo and 103 proteins in *Arabidopsis* was used to construct the neighbor-joining Tree. **b** A total of 25 open reading frame sequences in Lei bamboo and 103 proteins in *Arabidopsis* was used to construct the maximum likelihood tree

From the conserved 103 *Arabidopsis* MADS proteins in the hidden Markov model [40], we acquired 25 Lei bamboo transcriptome ORF sequences designed as MADS genes. The phylogenetic trees were constructed by using the NJ (Fig. 6a) and ML (Fig. 6b) methods based the MADS protein classification of *Arabidopsis* [41]. In the NJ and ML trees, a bit interior branches exist some minor differences, but in general, the classification results are very similar. It suggests that most proteins have similar origin in both trees. Here, we used NJ phylogenetic tree for further analysis. Accordingly, 11 unigenes were found to belong to the Mα, Mβ and Mγ, two were grouped in the Mδ type and other 12 were in the MIKC type.

**Fig. 7:**
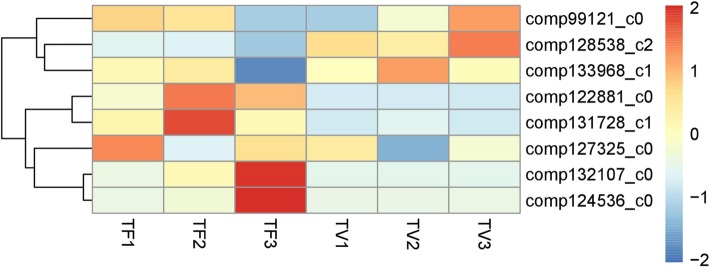
The heatmap of the MADs genes belong to ABCDE model in Lei bamboo

According to the phylogenetic tree (Fig. 6a), we found eight unigenes belonging to ABCDE model genes. A class genes represented by *APETALA1(AP1)* and *APETALA2(AP2)* in *Arabidopsis* [42], and three unigenes (*comp128538_c2_orf1*, *comp133968_c1_orf1*, *comp127325_c0_orf1*) were in a same group with them. *PI(PISTILLATA)* and *AP3(APETALA3)* belong to B class genes in *Arabidopsis* [43], while there are two unigenes (*comp132107_c0_orf1*, *comp99121_c0_orf1*) belonging to B class genes. In *Arabidopsis*, *AG(AGAMOUS)* is recognized as C genes, *STK* is recognized as D genes, they have similar expression patterns [44–46], but we identified only one gene (*comp124536_c0*) belonging to the C/D genes family. The representative gene of the E class gene is *SEP(SEPALLATA)1/2/3/4* [47], and two unigenes (*comp122881_c0_orf1*, *comp131728_c1_orf1*) were found to belong to E class genes. According to RNA-seq results, eight genes showed different patterns of expression in different samples (Fig. 7). In these, *comp99121_c0*, *comp128538_c2* and *comp133968_c1* were highly expressed in TV stages while the rest four genes (comp132107_c0, comp124536_c0, comp122881_c0, comp131728_c1) that belong to B, C/D and E clades were up-regulated significantly in TF stages, suggesting the essentials of B, C/D and E genes in flower organ development of Lei bamboo. Moreover, the *comp119979_c0_orf1* and *comp127580_co_orf1* were identified to belong to *SVP* (*SHORT VEGATATIVE PHASE*), while *comp131813_co_orf1* was belonged to *SOC1* (*SUPPRESSOR OF OVEREXPRESSION OF CO1*), indicating their functional roles as flower regulators in plants [48–50].

### Verification of the gene expression by qRT-PCR

**Fig. 8:**
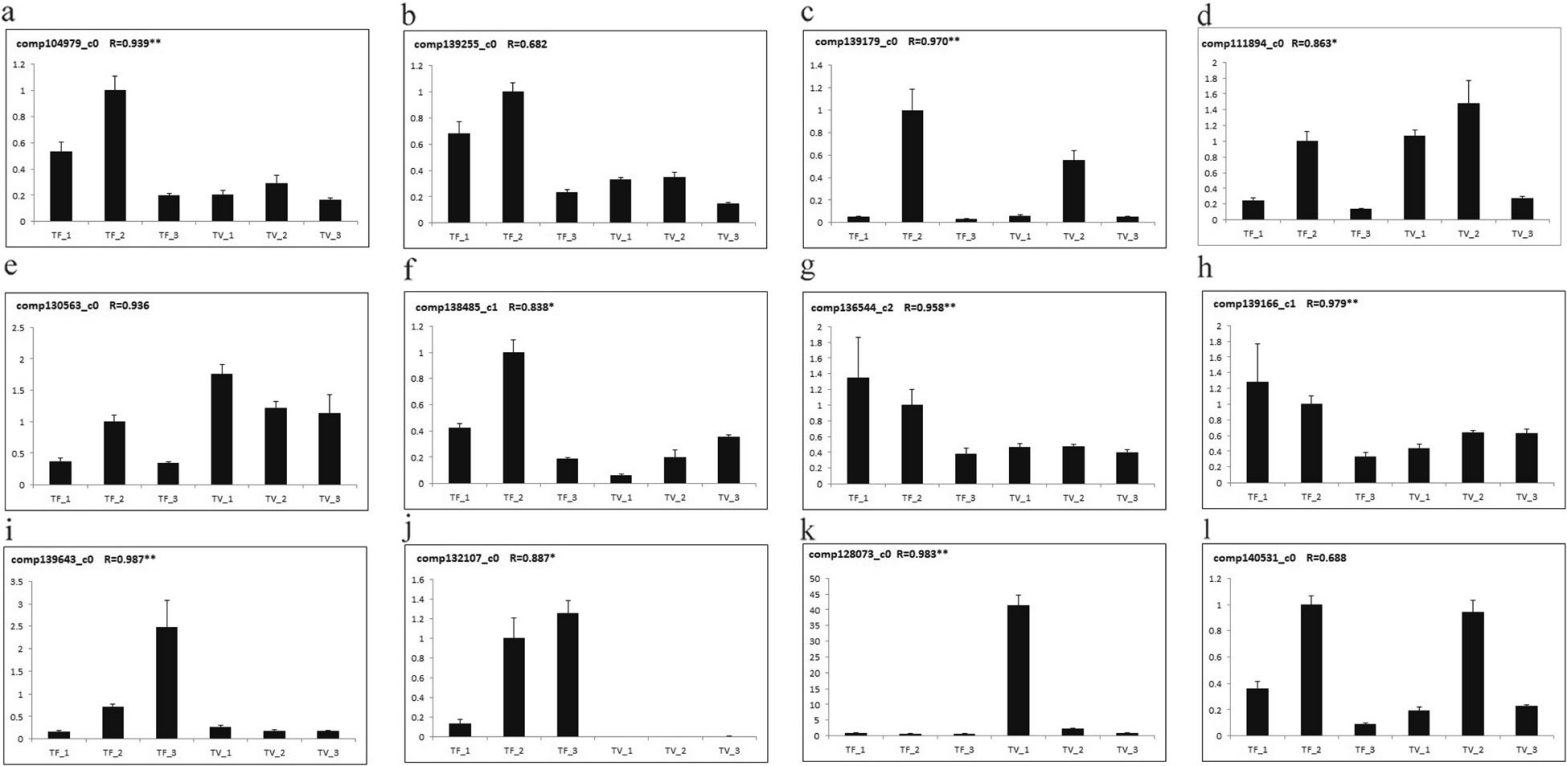
Quantitative real-time RT-PCR confirmation of 12 candidate genes at the six stages

Transcriptional regulation revealed by RNA-seq was confirmed in a biologically independent experiment using RT-PCR, a total of 12 genes were chosen to design gene-specific primers (Additional file 5). Finally, 10 genes (*comp104979_c0*, *com*p*139179_c0*, *comp111894_c0*, *comp130563_c0*, *comp139255_c0*, *comp136544_c2*, *comp139166_c1*, *comp139643_c0*, *comp132107_c0* and *comp128073_c0*) showed significant correlations (*P* = 0.05 & *P* = 0.01) between the qRT-PCR data and the RNA-seq results (Fig. 8), suggesting a good reproducibility between transcript abundance assayed by RNA-seq and the expression profile revealed by qRT-PCR.

## Discussion and conclusion

As the randomness and uncertainty of bamboo flowering, how to obtain the accurate flower samples is the most crucial problem for bamboo flowering mechanism study. Here, the sampling time of bamboo flowering materials was earlier than that in most usual researches. For example, the moso bamboo flower buds were took when the flower morphology has appeared [51], but we pre-judged and began to collect the samples in earlier stages (TF_1 and TV_1) when the floral or leaf buds have no obvious different phenotype. This method makes it better to identify the flower induction related genes in bamboo. Moreover, our sampling method is “one-to-one” pattern. As the development of vegetative buds and stems may accompany the flowering process of bamboo, the flower development samples and corresponding vegetative samples were “co-sampled” and “co-sequenced” in our study to better ensure the same genetic background (Fig. 1g). It is not only beneficial to avoid interference from vegetative growth related genes in bamboo flowering mechanism study, but also helpful to identify the related genes involved in vegetative periods in Lei bamboo. Above all, our research will provide a new sampling method for studying the flowering mechanism of bamboo.

We obtained 132,678 assembled unigenes using RNA-seq, with 7266 DEGs identified between flowering and vegetative buds at the three stages of Lei bamboo. Although the RNA-seq databases of certain bamboo species, such as moso bamboo, Ma bamboo (*Dendrocalamus latiflorus*) and *Bambusa edulis* [52–54], have been established, we provided transcriptome data in Lei bamboo for the first time. Finally, a number of DEGs were determined according to the expression profiles, indicating their functional roles in early flowering initiation and flower development of Lei bamboo. For example, in the TF_1 stage, the “detection of abiotic stimulus”, “detection of external stimulus”, “detection of light stimulus” genes were found to be enriched. But the “photoperiod” genes were downregulated compared with TV_1. Interestingly, the stress-responsive genes were also identified to be highly-expressed in moso bamboo, but the genes employed in typical flowering promotion pathways (such as those in the photoperiod, gibberellins, ambient-temperature or light-quality pathways) and floral pathway integrator (FPI) genes were expressed lowly in these floral tissues [52]. It is highly likely that the flowering of bamboo associated more with the environmental stress factors than photoperiod or other traditional pathways. Plant hormones play necessary role during flower development [47–51]. In Lei bamboo, the “plant hormone signaling transduction” related genes were abundant in TF_1 compared with TV_1. In previous study, the endogenous hormone concentrations were found to be changed with the development of flower bud morphological differentiation [55]. It seems that the auxin (IAA) and Cytokinine (CTK) are act as promoters during flower bud differentiation [56]. As we known, *AUX/LAX* genes encode auxin influx transporters that have distinct developmental functions and regulatory mechanisms in *Arabidopsis*, while CTK regulates the activity of reproductive meristems, flower organ size, ovule formation, and thus seed yield [57]. Here, the DEGs *CRE1* (*comp138523_c0*) and *ARR-B* (*comp139166_c1* and *comp134657_c0*) enriched in the CTK pathway and *AUX1/LAX* (*comp138485_c1, comp137757_c1*), *TIR1*(*comp136544_c2*) and *ARF* (*comp139738_c1*) enriched in the auxin pathway were all significantly upregulated in TF_1 stage. It implies the essential roles of these CTK and IAA related genes in Lei bamboo flower induction.

In TF_3, some “female gamete generation”, “flower development”, “pigment biosynthetic process”, “anther morphogenesis” related genes were up-regulated significantly compared with vegetative stage (TV_3). For higher plants, the anther and carpel developments were recognized as the most important stages of organogenesis regarding sexual reproduction processes [46]. Therefore, these DEGs might have essential roles in the sextual reproduction process of Lei bamboo. The family of MADS-box genes play important roles in plant flower development. They are expressed regionally specific in particular flower tissues, during differentiation growth of the flower development. According to previous reports, MADS-box genes participate in floral organ development and flowering time regulation [58]. The MIKC-type proteins represent a class of MADS-box transcription factors, and the MIKC genes could control flowering time and flower organ identity, especially ABC model genes [59, 60]. In rice, 34 MADS-box genes have been identified, and 15 of which regulate flower development [61]. In moso bamboo, 34 MADS-box genes were identified and the expression patterns of most genes were found to similar to *Arabidopsis* and rice [23]. In our study, we found eight MADS box genes belonging to ABC model genes. Combing with the expression profiles, the B, C/D and E genes should be associated more with flower development than A genes. In *Arabidopsis*, B genes(*PI* and *AP3*) could control petal identity with A class genes, and together with C class genes form the stamen [47]. We found *comp132107_c0* have high expression especially in maturation process of floral organs, hence we infer that *comp132107_c0* might mainly control petal and stamen identity in later floral stages. Additionally, it is generally accepted that the C class genes (*AG*) control the development of stamen and pistil [45], and the D class genes are involved in ovule identity [11]. But in petunia, D genes are not essential to confer ovule identity, instead, this function is redundantly shared among all AG members, and D genes might participate in floral determinacy [62]. In our study, *comp124536_c0* had high expression in maturation of floral organs, so it might be a key gene involved in floral determinacy in later development stages. The E genes were known to participate in regulation of flower organ characteristics [63]. *SEP 1/2/3/4* are key genes in floral organ development, and their functions are redundant in floral identity. These genes play fundamental roles by interacting with other MADS-box genes products [47, 64, 65]. Two genes *comp122881_c0* and *comp131728_c1* had high expressions in flower plants and low expressions in non-flower plants, so they might exhibit similar functions as the *SEP1/2/3/4* gene in Lei bamboo. The ABCDE class MADS-domain transcription factors can assemble into an organ-specific quaternary protein complex to control downstream genes [58]. Whether this interaction is suitable for Lei bamboo needs to be further explored.

## Supplementary information

**Additional file 1.** Summary of the annotations for the assembled unigenes in public databases.

**Additional file 2.** Cluster analysis of differentially expressed genes. (a) Heat map of DEGs expression. TF_1: the expression value in TF_1; TF_2: the expression value in TF_2; TF_3: the expression value in TF_3; TV_1: the expression value in TV_1; TV_2: the expression value in TV_2; the expression value in TV_3; TF_12: average of the expression value in TF_1 and TF_2; TF_13: average of the expression value in TF_1 and TF_3; TF_23: average of the expression value in TF_2 and TF_3; TV_12: average of the expression value in TV_1 and TV_2; TV_13: average of the expression value in TV_1 and TV_3; TV_23: average of the expression value in TV_2 and TV_3; TF: average of the expression value in TF_1, TF_2 and TF_3; TV: average of the expression value in TV_1, TV_2 and TV_3. Expression values for 6 libraries are presented as RPKM normalized by transformed counts. (b) Six main clusters are shown based on the heat map of DEGs.

**Additional file 3.** KEGG pathways and the corresponding unigene numbers and IDs in the transcriptome of Lei bamboo.

**Additional file 4.** Summary of the GO enrichment analysis of the DEGs in each comparison.

**Additional file 5.** Primer sequences for qRT-PCR analysis.

## Data Availability

All data generated or analyzed during this study are included in this published article。.

## Abbreviations

DEGs: Differentially expressed genes
GO: Gene ontology
KEGG: Kyoto encyclopedia of genes and genomes
KO: KEGG ortholog database
COG: Clusters of orthologous groups of proteins
Nr: NCBI non-redundant protein sequences
Nt: NCBI non-redundant nucleotide sequences
Pfam: Protein family
RPKM: Reads Per Kilobase per Million mapped reads
qRT-PCR: quantitative real-time PCR
Swiss-Port: A manually annotated and reviewed protein sequence database
TF: Flowering buds
TF_1: Flowering buds of formation of inflorescence meristem
TF_2: Flowering buds of formation and identity determination of floral organs
TF_3: Flowering buds of maturation of floral organs
TV: Vegetative buds
TV_1: Vegetative buds from a non-flowering bamboo plant growing on the same rhizome of the flowering bamboo plant were sampled at the same time with TF_1
TV_2: Vegetative buds from a non-flowering bamboo plant growing on the same rhizome of the flowering bamboo plant were sampled at the same time with TF_2
TV_3: Vegetative buds from a non-flowering bamboo plant growing on the same rhizome of the flowering bamboo plant were sampled at the same time with TF_3
